# Microstructure of TiAl Capsules Processed by Electron Beam Powder Bed Fusion Followed by Post-Hot Isostatic Pressing

**DOI:** 10.3390/ma16165510

**Published:** 2023-08-08

**Authors:** Hanieh Bakhshi Farkoush, Giulio Marchese, Emilio Bassini, Alberta Aversa, Sara Biamino

**Affiliations:** 1 DISAT—Department of Applied Science and Technology, Politecnico di Torino, Corso Duca degli Abruzzi 24, 10129 Torino, Italy; giulio.marchese@polito.it (G.M.); emilio.bassini@polito.it (E.B.); alberta.aversa@polito.it (A.A.); sara.biamino@polito.it (S.B.); 2 IAM@PoliTo—Interdepartmental Center of Integrated Additive Manufacturing, Politecnico di Torino, Corso Castelfidardo 51, 10129 Torino, Italy; 3 INSTM—Consorzio Interuniversitario Nazionale per la Scienza e Tecnologia dei Materiali, Via G. Giusti 9, 50121 Firenze, Italy

**Keywords:** titanium aluminide, PPBs, microstructure, EB-PBF, HIP

## Abstract

The microstructures of intermetallic γ-titanium aluminide (TiAl) alloys are subjected to a certain degree of Al evaporation when processed by electron beam powder bed fusion (EB-PBF). The magnitude of the Al-loss is mainly correlated with the process parameters, and highly energetic parameters produce significant Al evaporation. The Al-loss leads to different microstructures, including the formation of inhomogeneous banded structures, thus negatively affecting its mechanical performance. For this reason, the current work deals with creating EB-PBFed TiAl capsules with the inner part produced using only the pre-heating step and melting parameters with low energetic parameters applying high beam speed from 5000 to 3000 mm/s. This approach is investigated to reduce the Al-loss and microstructure inhomogeneity after hot isostatic pressing (HIP). The results showed that the HIP treatment effectively densified the capsules obtaining a relative density of around 100%. After HIP, the capsules produced with the inner part melted at 3000 mm/s presented a lower area shrinkage (around 6.6%) compared to the capsules produced using only the pre-heating step in the core part (around 20.7%). The different magnitudes of shrinkage derived from different levels of residual porosity consolidated during the HIP process. The HIPed capsules exhibited the presence of previous particle boundaries (PPBs), covered by α_2_ phases. Notably, applying low energetic parameters to melt the core partially eliminates the particles’ surface, thus reducing the PPBs formation. In this case, the capsules melted with low energetic parameters (3000 mm/s) exhibited α_2_ concentration of 3.5% and an average size of 13 µm compared to the capsules produced with the pre-heating step in the inner part with an α_2_ around 5.7% and an average size around 23 µm. Moreover, the Al-loss of the capsules was drastically limited, as determined by X-ray fluorescence (XRF) analysis. More in detail, the capsules produced with the pre-heating step reported an atomic percentage of Al of 48.75, while using low energetic melting parameters led to 48.36. This result was interesting, considering that the massive samples produced with standard parameters (so more energetic ones) revealed atomic Al percentage from 48.04 to 47.70. Finally, the recycled small particles showed a higher fraction of α_2_ phases with respect to the coarse particles, as determined by X-ray diffraction (XRD).

## 1. Introduction

Intermetallic γ-titanium aluminide (TiAl) based alloys are excellent candidates for working at elevated temperatures (around 600–750 °C) due to their low density, high specific strength, good oxidation, and creep resistance. These properties make the intermetallic TiAl alloys attractive for aerospace and aeronautical applications. For instance, low- pressure turbine blades are already produced in TiAl alloys, resulting in less fuel consumption than heavy Ni-based superalloys characterized by a density around double with respect to the TiAl alloys [1,2,3,4].

The TiAl alloys are traditionally mainly processed by casting (e.g., investment casting), but in the last years, the additive manufacturing electron beam powder bed fusion (EB-PBF) has also gained attention due to the possibility of creating crack-free complex shape parts in a single step [5,6,7]. The EB-PBF process uses a defocused electron beam to heat the building platform in order to reach an initial high pre-heating temperature (around 1050–1100 °C for TiAl alloys, which is over the brittle-to-ductile transition temperature of around 700 °C) inside the chamber, operating under vacuum. The subsequent steps are commonly the following: (a) pre-heating the layer of powder using a defocused electron beam to partially sinter the powder and avoid the smoke phenomenon due to the accumulation of the electrical charge on the surface of the particles; (b) the melting step consisting of a focused electron beam to selectively melt the powder on the building platform; (c) possible post-heating step can be applied to keep high the temperature of the building platform based on the material; and (d) the building platform is lowered, and a new layer of powder is spread on the building platform, and subsequently the process is repeated from “a” to “d”. Moreover, a reduced helium pressure is used to keep the overall vacuum level in the chamber at 2 × 10^−3^ mbar, thus reducing the risk of electrostatic charging and, therefore, possible smoke phenomenon. After the job, the sintered powder can be collected in a sand-blasted machine, and then the used powder can be sieved for subsequent job productions [8,9,10].

However, the process parameters such as the beam current, beam speed, line offset, and melting strategy can trigger a certain level of Al evaporation during the production of the TiAl components. The TiAl alloys commonly can present inhomogeneous microstructures consisting of banded structures made up of altered Al-depleted and Al-rich zones, thus leading to variation in the mechanical properties [5,11,12,13]. The Al evaporation can be mitigated by the application of low energetic parameters, although low melting parameters trigger the formation of large defects (e.g., lack of fusion) [14]. The presence of these defects can drastically limit the mechanical properties of the components. It is, therefore, fundamental to design strategies to mitigate the Al-loss and to keep a high densification level during the TiAl production through the EB-PBF process [15,16]. In order to consolidate the defects, the hot isostatic pressing (HIP) process can be employed [1,15,16]. Actually, HIP treatment is mandatory for critical components (e.g., critical aerospace parts) to consolidate the materials, removing defects, like pores and lack of fusion. However, an inhomogeneous microstructure in the as-built state can also remain after the HIP treatment; thus, explaining why it is crucial to perform the post-heat treatments starting from a homogenous microstructure [17,18].

In fact, heat treatments are commonly used for additively manufactured materials to improve the material’s mechanical performance [17,19,20].

Regarding the TiAl alloys, the material can be annealed at a temperature over the alpha transus temperature followed by rapid cooling to generate a fully lamellar microstructure. On the contrary, decreasing the annealing temperature below the alpha transus temperature makes it possible to generate near lamellar, duplex, near gamma, and equiaxed grains. A microstructure composed of lamellar grains offers high creep resistance and toughness fracture, although it reduces the ductility at room temperature and fatigue resistance. Conversely, equiaxed grains generate higher ductility at room temperature and fatigue resistance combined with reduced creep resistance and fracture toughness. It is, therefore, possible to play with the heat treatments to design the fraction of lamellar and equiaxed grains to obtain a desired combination of mechanical properties [21,22,23]. However, the presence of an altered microstructure can reduce mechanical performance and promote the premature formation of cracks inside the components.

Another strategy recently proposed in the literature to improve the microstructure homogeneity of the TiAl alloys has been the fabrication of near net shape close capsules filled with sintered powder by means of the EB-PBF and then post-HIPed. A similar approach should eliminate the issue of the Al-loss and could bring a more homogenous microstructure. These studies showed the possibility of obtaining the densification of the capsules, although microstructures inhomogeneity remained like the formation of previous particle boundaries (PPBs). These microstructure features can decrease the mechanical performance of the components and should be eliminated [15,16].

The purpose of the current work is to investigate the densification level and microstructure homogeneity of capsules made of TiAl filled with sintered powder due to the pre-heating step and capsules produced with a fast melting step in the inner part after post-HIP treatment. Therefore, this work aims at investigating a possible strategy to obtain a homogenous microstructure of the HIPed EB-PBFed TiAl alloy. This is a fundamental step to guarantee a homogenous microstructure, and, therefore, constant mechanical performance also after the application of a subsequent heat treatment.

## 2. Materials and Methods

The current work employed gas-atomized Ti-48Al-2Nb-2Cr powder supplied by Arcam EBM (Arcam, Mölnlycke, Sweden), a GE Additive group. The particle size distribution range from 40 to 150 µm and the chemical composition determined by optical emission spectrometer-inductively coupled plasma (OES-ICP) is reported in Table 1.

**Figure 1:**
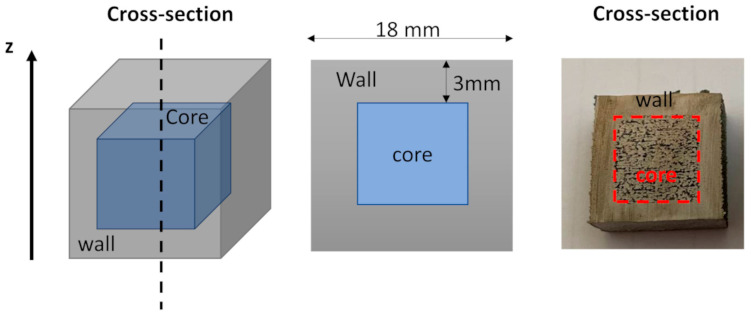
Schematic illustration of the capsule specimens. The wall and core are processed using different process parameters.

Capsule samples of 18 × 18 × 18 mm^3^ were produced using an Arcam A2X machine (Mölnlycke, Sweden) with a maximum electron beam power of 3 kW. For the capsules, the outer part (3 mm) denominated wall was produced using parameters to obtain a highly dense part, while the inner part (denominated core) was processed using low energetic parameters, as schematically reported in Figure 1. Moreover, some capsules were processed without the melting step inside the core part. The wall and core presented an overlapping of 1 mm.

Two capsules were produced for each combination of process parameters reported in Table 2, using a scanning strategy with 90° rotation. Note that two capsules were produced without the melting step in the inner part, thus avoiding the melting of the powder. The pre-heating parameters were set to obtain a temperature of around 1050 °C during the building production. The parameters used for the wall are standard process parameters that guarantee a high densification level, as reported in a previous study by some of the authors [24]. On the contrary, the melting parameters of the core part were selected to use higher beam speed, lower beam current, and larger line offset in order to generate lower area energy and consequently induce only limited densification and Al evaporation.

Finally, the capsule samples were compared to massive samples produced with the parameters of the wall part, employing different focus offset sets of 10 mA and 30 mA. The focus offset affects the spot size of the electron beam, thus, focus offset 10 mA produces a narrower spot size than focus offset 30 mA [24].

Some specimens were subjected to HIP treatment using a Quintus QIH15L machine. The HIP treatment parameters were a temperature of 1260 °C, a pressure of 170 MPa, and a dwell time of 4 h, followed by furnace cooling. These parameters were already employed in a previous study by some of the authors [1]. The HIP treatment commonly consolidates the material removing critical defects and promoting the microstructure evolution [1,15].

The capsules in the as-built and HIPed state were cut along the building direction and then grounder using SiC papers up to 2500 grit, polished with a diamond suspension of 3 µm, and finally polished with a 0.03 µm silica suspension. In order to assess the residual porosity, the samples were examined by means of a light optical microscope (LOM, Leica DMI 5000 M, Wetzlar, Germany). Fifteen images per sample were acquired at 50× magnification and analyzed using ImageJ (https://imagej.net/ij/, accessed on 13 July 2023), an open-source image analysis software.

The HIPed capsules were also analyzed to investigate their microstructure evolution and the level of microstructure homogeneity. For microstructural analysis, the samples were etched with Kroll’s etchant for 7–10 s. The etched samples were analyzed by LOM and Scanning Electron Microscope (SEM), using a Phenom Pro X (ThermoFisher, Waltham, MA, USA) equipped with an Energy-Dispersive X-ray spectrometry (EDS) detector. Moreover, the microstructure of the polished samples was analyzed by means of SEM TESCAN S9000G (Tescan Group, Brno, Czech Republic) equipped with electron backscattered diffraction (EBSD) using a tilting angle of 70° and scanned at 20 kV and 10 nA. A step size of 0.8 µm was used for performing the EBSD analysis at a magnification of 500×. The Al evaporation of the capsules and massive samples was analyzed by means of X-ray fluorescence (XRF), reporting the average value and standard deviation obtained by three measurements on each sample.

After the EB-PBF production, the used powder was collected and divided into three categories of particle size distribution using sieves. Small particles (≤53 µm), medium particles (from 53 to 106 µm), and large particles (≥106 µm). These three categories of particle size distribution were analyzed by X-ray diffraction to evaluate the presence of the phases. X-ray diffraction (XRD) tests were performed using an XRD diffractometer (X-Pert Philips diffractometer, PANanalytical, Almelo, The Netherlands) by CuKα radiation in a Bragg Brentano configuration working at 40 kV and 40 mA with a step size of 0.013° and counting at each step for 25 s.

**Figure 2:**
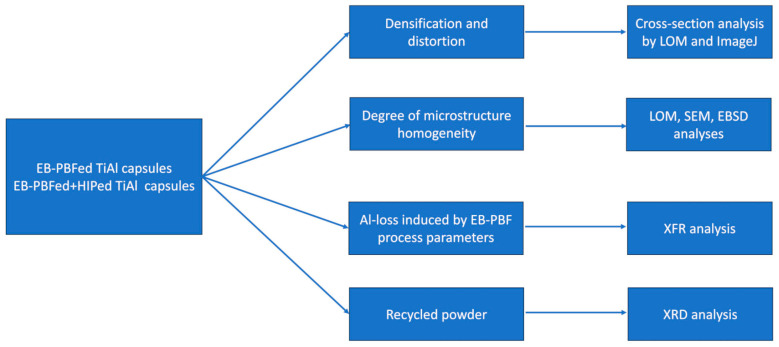
Characterization steps of the EB-PBFed and EB-PBFed + HIPed capsules to determine the densification and distortion, degree of microstructure homogeneity, Al-loss, and impact of the EB-PBF process on the recycled powder.

Overall, the characterization steps of the EB-PBFed and HIPed capsules are illustrated in Figure 2. The densification and distortion induced by the HIP treatment were determined by cross-section analysis using LOM. Afterward, the magnitude of microstructure homogeneity was investigated by the combination of LOM, SEM, and EBSD analyses. The Al evaporation induced by the EB-PBF process was also checked by XRF analysis. Finally, the used powder was analyzed by XRD to highlight the possible effect of the pre-heating step on the microstructure of the powder.

## 3. Results and Discussion

### 3.1. Porosity and Distortion Evaluation

**Figure 3:**
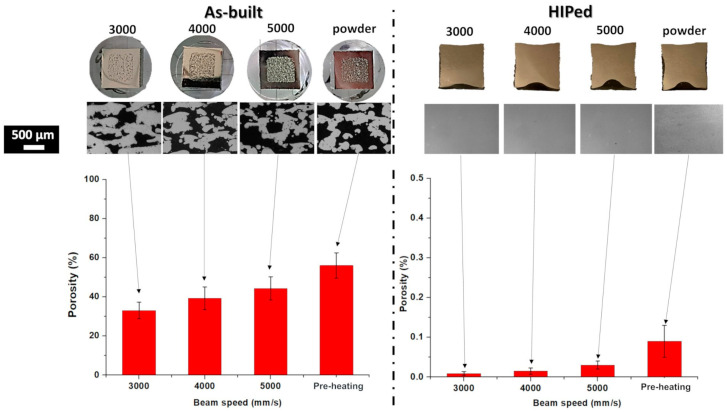
Image, LOM images, and porosity level of the capsule samples processed using different parameters in the as-built and HIPed states.

The cross-section of the capsules built with different process parameters in the as-built and HIPed states are provided in Figure 3. In the case of the as-built capsules, the images and LOM images revealed an increment of the porosity with the acceleration of the beam speed, reaching 56.0% in the case of the capsule without the melting step.

The addition of the melting step provides a partial melting of the particles, and reduced porosities of around 44.3%, 39.2%, and 32.9% were generated by a gradual reduction of the beam speed from 5000 to 4000 and finally 3000 mm/s. The low densification level is attributed to the low energetic parameters employed to limit the possible microstructure inhomogeneity in the inner part. It should be noted that the level of residual porosity of the capsules with only inner pre-heating is in agreement with the work of Bieske et al. [15].

After HIP, the images and LOM micrographs of the core part of the capsules showed the absence of large pores, thus indicating that the HIP parameters effectively densify the samples for all the conditions. For the analysis, there is a trend of a slight porosity increment with the increment of the beam speed. The porosity presented values of around 0.009% for 3000 mm/s and then slightly increased to 0.015% and 0.03% for 4000 and 5000 mm/s. Finally, the porosity was around 0.09% for the capsule produced only with the pre-heating step in the core part. However, considering the very low level of residual porosity of the HIPed capsules, the core density can be indicated as fully densified as 100%.

**Figure 4:**
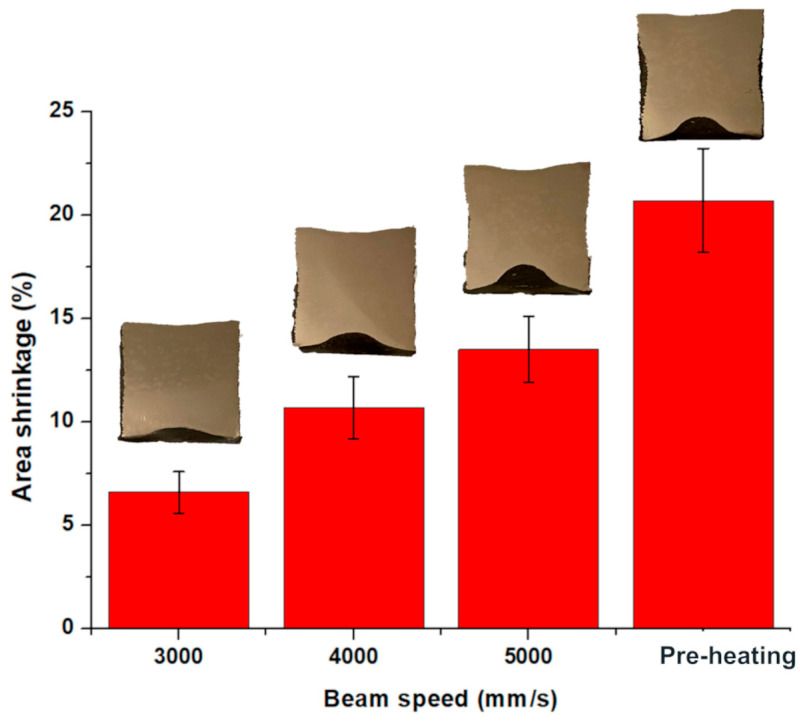
Area shrinkage of HIPed capsules processed using different process parameters.

The HIPed capsule presented an area shrinkage due to the consolidation of the inner part characterized by partially melted or not melted powder, as depicted in Figure 4. The shrinkage increased with the acceleration of the beam speed, reaching the highest magnitude for the capsule produced without the melting step. The lowest shrinkage was recorded as 6.6% for a beam speed of 3000 mm/s, while the highest shrinkage was recorded as 20.7% for the capsule without the melting step. The results agree with the level of porosity detected in the as-built capsules. In fact, the higher the porosity level, the greater the shrinkage in the capsule under HIP treatment.

The capsules produced with a beam speed of 3000 mm/s (abbreviated as capsule-3000) and the capsules produced without an inner melting step (abbreviated as capsule-preheating) were compared to reveal their microstructure.

Considering that it is crucial to know the area shrinkage after HIP for designing the dimensions of the parts, these results indicated that the capsule-3000 reduces the overestimation of the part dimensions in the as-built state compared to the capsule-pre-heating.

Note that the distortion was not homogenous on all the sides of the capsule because the side attached to the building platform resulted slightly reduced (around 2.8 mm instead of 3 mm) due to the interaction with the building platform, and consequently, the shrinkage is more significant along that side. For this reason, it was evaluated the area shrinkage, which takes into account the global deformation of the components.

Based on the results, designing and obtaining a homogenous thickness for the borders of the capsules is essential to eliminate preferential side distortions that can occur on the thinner sides as well as it is fundamental to optimize the thickness of the borders of the capsules. Moreover, a small thickness composed of superficial defects reaching the inner part may promote argon flow during HIP treatment, thus suppressing capsule densification. On the other hand, too thick borders could increase the overstock of material. Finally, a machining step can be performed to remove the wall structures in order to have components with the same microstructure features.

### 3.2. Microstructure Evolution

**Figure 5:**
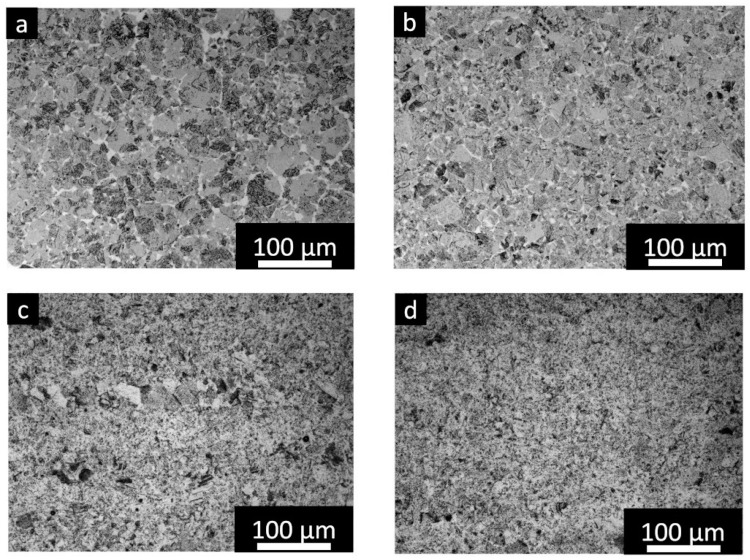
LOM images of HIPed TiAl samples: (**a**) capsule-pre-heating; (**b**) capsule-3000; (**c**) massive sample with a focus offset of 10 mA; and (**d**) massive sample with a focus offset of 30 mA.

Figure 5a,b shows the microstructure of the inner part of the capsule consisting of γ+ α_2_ grains after the HIP treatment. As visible in the case of the HIPed capsule-pre-heating (Figure 5a), previous particle boundaries (PPBs) are derived from the powder, showing α_2_ phases generated to the surface of the original particles. On the other hand, using low energetic melting parameters apparated to limit the presence of the PPBs (Figure 5b), as visible in the HIPed capsule-3000 sample. In this case, the sample reveals α_2_ phases with smaller dimensions and better distributed throughout the material.

The current results on the capsule-pre-heating are in agreement with other studies revealing that the HIPed capsules exhibited the formation of PPBs due to the Al evaporation occurring during the pre-heating step at the surface of the particles, thus indicating that the pre-heating step must be tailored to reduce the Al evaporation [15,16]. The PPBs formation in the case of EB-PBF followed by HIPed is different with respect to the traditional HIPed TiAl powder in the powder metallurgy, where the surface enrichment of oxygen tends to stabilize the formation of α_2_ phase under heat treatment. Likewise, the formation of PPBs is detrimental to the mechanical properties, reducing the ductility and mechanical properties in temperatures, and therefore, their presence must be suppressed to guarantee high mechanical performance [25,26,27].

It is interesting to compare the microstructure of the HIPed capsules with two massive HIPed TiAl samples produced using different focus offset values. Note that the HIP parameters are the same as the capsule and that the TiAl massive samples produced with two focus offset values are reported in a previous work of some of the authors [24]. The samples produced using a focus offset of 10 mA (Figure 5c) exhibited banded microstructure, thus indicating an intense Al evaporation. On the other hand, the samples produced with a focus offset of 30 mA (Figure 5d) showed a homogenous microstructure without banded structures, thus indicating a limited Al-loss derived from the melting step. In this case, the microstructure variation is derived from the different spot sizes of the electron beam, which involves a different melt pool size. For the focus offset of 10 mA, the spot size is smaller, thus generating a depth and narrow melt pool leading to more Al evaporation, while for the focus offset of 30 mA, the spot size is larger, thus creating a more shallow and large melt pool mitigating the Al evaporation.

Overall, there were no banded structures in the HIPed capsule samples, but there was a high frequency of PPBs in the HIPed capsule-pre-heating and still a moderate quantity of PPBs in the HIPed capsule-3000.

**Figure 6:**
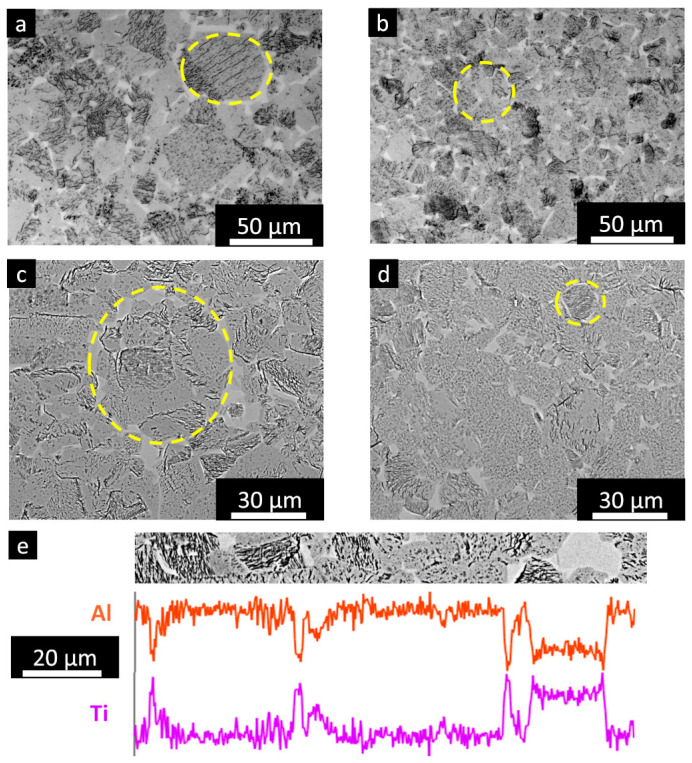
LOM images at high magnification of HIPed: (**a**) capsule-pre-heating; (**b**) capsule-3000; SEM image of (**c**) capsule-pre-heating; (**d**) capsule-3000; and (**e**) SEM + EDS scan line showing that the bright phases are α_2_ phase enriched in Ti and depleted of Al. Some PPBs are pointed out by yellow circles in the LOM and SEM images.

The HIPed capsule-pre-heating presented γ grains ranging from 70 to 10 µm with an average grain size of around 60 µm, while the HIPed capsule-3000 exhibited γ grains with dimensions from 50 to 6 µm with an average grain size of around 44 µm, as depicted in Figure 6a,b, respectively. Regarding the α_2_ phase, the HIPed capsule-pre-heting revealed sizes from a few microns up to around 30 µm with an average size of around 23 µm, while the HIPed capsule-3000 sample exhibited size between a few microns and 25 µm with an average size of around 13 µm.

For the microstructure observations, the HIPed capsule-pre-heating sample presented larger grains compared to the HIPed capsule-3000 samples. Moreover, the HIPed capsule-pre-heating exhibited a higher number and bigger PPBs with respect to the HIPed capsule-3000, as pointed out by the cycles in Figure 6a–d.

It is, therefore, visible that melting the internal core of the capsule with low energetic parameters followed by HIP treatment involved smaller grains and low concentration of PPBs. The SEM + EDS scan line confirmed that the bright phase is the α_2_ phase due to the enrichment in Ti and depletion in Al (Figure 6e).

Considering the TiAl phase diagram, the employed HIP treatment temperature (1260 °C) occurred in the zone of the α + γ phases below the alpha transus temperature, and the Al evaporation shifted the phase diagram to a zone with a higher quantity of α_2_ (at the same temperature) [4,22,23]. This result is correlated with the microstructure observation showing more intense α_2_ concentration close to the surface of particles, where the pre-heating step already provided Al evaporation.

**Figure 7:**
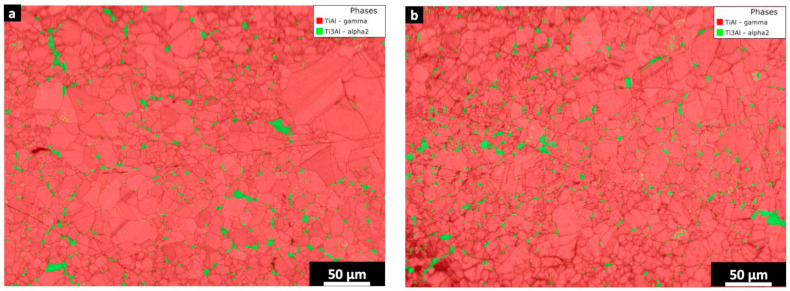
EBSD phase map of (**a**) HIPed capsule-pre-heating and (**b**) HIPed capsule-3000. The red represents the γ-TiAl phase, while the green indicates the α_2_ phase. Some PPBs are pointed out by yellow circles in the EBSD maps.

The EBSD maps of the HIPped capsule-pre-heating (Figure 7a) and HIPed capsule-3000 (Figure 7b) showed the presence of the γ phase as well as the α_2_ phase. The HIPed capsule-pre-heating exhibited larger dimensions and a higher α_2_ concentration of 5.7 ± 1.2% compared to the HIPed capsule-3000 sample with a concentration of 3.5 ± 0.3%.

The EBSD analysis confirms that in the HIPed capsule-pre-heating sample, the α_2_ phase tends to form along the surface of the particles due to the Al depletion that occurred in the pre-heating step, forming the PPBs as pointed out by the yellow circles. On the other hand, the PPBs formation is mitigated in the case of the HIPed capsule-3000 sample.

It is possible to infer that when the beam melts the powder inside the capsule, it breaks the powder’s surface, mixing the surface with the inner part of the particle in the melting stage, thus reducing the risk of forming PPBs particles. Conversely, the HIPed capsule-pre-heating sample is only subjected to a pre-heating step into the core part of the capsule, thus causing an alteration of the Al content between the surface of the particle and its core, developing more α_2_ phase decorating the surface of the particles under the temperature of the HIP treatment.

### 3.3. Al-Loss Evaluation

**Figure 8:**
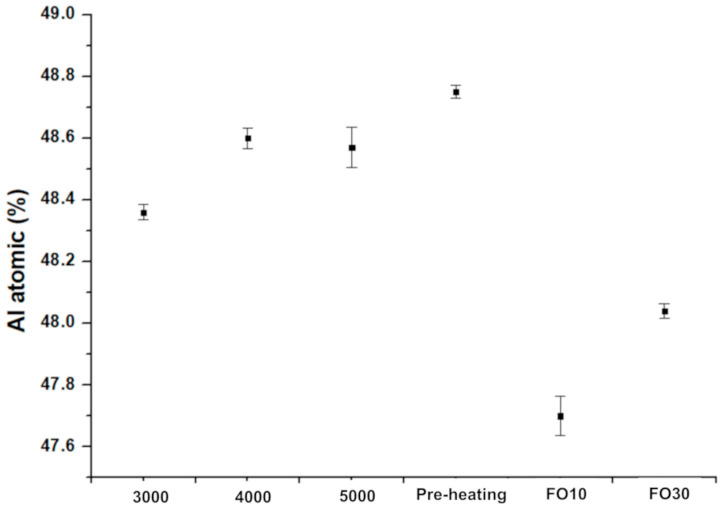
Al atomic percentage of the capsules and massive samples produced with different parameters by XRF analysis.

The capsule-pre-heating presented the highest Al content due to the limited effect of the pre-heating step in the Al depletion on the surfaces’ particles, as displayed in Figure 8. On the other hand, the melting step with high beam speed produces a progressive Al evaporation from 5000 to 3000 mm/s. Moreover, the HIPed capsule samples presented a less significant Al-loss than the massive samples produced with a focus offset of 30 mA and 10 mA.

The reduction of the beam speed involves higher energy density delivered on the powder, thus increasing the size and depth of the melt pool, consequently leading to more Al evaporation. On the other hand, low energetic melting parameters resulted in low Al-loss compared to the production of massive samples. Therefore, this approach seems interesting to avoid the issue of the massive samples, which could be characterized by a layered microstructure because of a significant Al-loss at the top of the melt pool.

However, one negative aspect of the HIPed capsule is represented by the PPBs that could alter the mechanical performance of the components. Therefore, in future works, the mechanical properties of capsule-3000 and massive samples produced with a focus offset of 30 mA will be evaluated and correlated with their microstructures to determine the magnitude of the impact of the PPBs.

### 3.4. XRD Analysis on the Powder

From the current microstructural analysis, small PPBs can still be detected in the HIPed capsule-3000 samples, while the large PPBs seemed suppressed by applying the melting step. This seems to suggest that the small particles presented a more pronounced Al-loss compared to the bigger particles.

**Figure 9:**
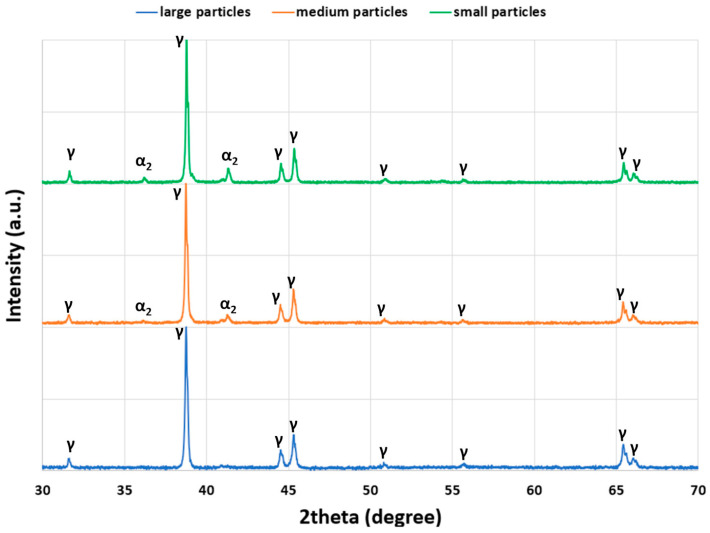
XRD patterns of the powder in three different particle size distributions: small particles (<53 µm), medium particles (from 53 to 106 µm), and large particles (>106 µm).

In order to confirm this hypothesis, XRD analysis was performed on the powder divided into three categories: small (<53 µm), medium (from 53 to 106 µm), and large (>106 µm), as displayed in Figure 9.

The three powder categories presented the peaks of the γ-TiAl phase, while the α_2_ phase showed a different trend. The small particles presented the highest concentration of α_2_ phase, while the medium particles exhibited a lower quantity, as denoted by the intensity of the peaks. Conversely, the large particles did not record the presence of the α_2_ phases, probably because their quantity was under the threshold of the XRD analysis.

This result supports the hypothesis that the small particles are subjected to more Al evaporation during the pre-heating step. Therefore, it is evident that using a melting step can help partially avoid the formation of PPBs, but it is still fundamental to tailor the pre-heating parameters to mitigate the superficial Al-loss occurring in this step.

A similar result must be taken into account to decide the maximum number of recycling of the powder. In fact, powder recycling could increment the α_2_ fraction on the particles, leading to more PPBs when the strategy of capsule production by EB-PBF is combined with HIP treatment to obtain dense samples. On the contrary, the production of massive samples using standard melting parameters could allow the use of the recycled powder more times since the melting parameters melt the particles, eliminating the risk of the PPBs formation even if the progressive Al-loss during pre-heating has to be taken into account.

## 4. Conclusions

The current work shows the development of TiAl capsules produced by EB-PBF and then post-processed by HIP treatment. Creating capsules with low energetic parameters in the core part seems helpful in mitigating the part distortion and PPBs formation after HIP treatment. The main conclusions can be drawn:

- Proper process parameters combined with the HIP treatment can produce dense samples with a relative density close to 100% both for the capsule with only the inner pre-heating step and the capsule with the inner melting step from 3000 mm/s to 5000 mm/s.
- The HIPed capsules showed a distortion due to the consolidation of the powder. The distortion can be limited by melting the inner part of the capsule with high beam speed (low energetic parameters). Using the current approach, area shrinkage of around 6.6% can be obtained in the case of capsule-3000.
- The application of high beam speeds to melt the internal part of the capsules limited the formation of large PPBs. In fact, the application of low energetic melting parameters partially melts the surface of the particles, reducing the risk of PPBs formation. The capsules-3000 showed an α_2_ concentration of 3.5% and an average size of 13 µm against the capsules-pre-heating with an α_2_ around 5.7% and an average size of around 23 µm.
- From the analysis, it seems that the particles with small sizes formed more α_2_ phases with respect to the bigger particles, thus generating more PPBs during the HIP treatment. Therefore, this aspect should be taken into account for considering the recycling of the powder. Significantly, the presence of small particles should be carefully checked.

Hence, the results indicate that producing massive samples tailoring the melting step in order to reduce the Al-loss at the top of the melt pools still represents the most suitable approach to achieve a homogenous microstructure. Although, the current study suggests that further exploration of the capsule strategy to mitigate the inhomogeneity could be conducted by tailoring the melting parameters in the capsule’s core.

## Figures and Tables

**Table 1:** Chemical composition of TiAl powders (at %) obtained by OES-ICP analysis.

Al	Cr	Nb	Fe	Ti
48.73	1.92	2.00	0.03	Bal.

**Table 2:** Process parameters employed for producing the capsule specimens. The constant parameters are the voltage of 60 kV, focus offset of 15 mA, and layer thickness of 90 µm with a scanning strategy of 90° rotation.

Samples	Beam Speed (mm/s)	Beam Current (mA)	Line Offset (mm)	Area Energy (J/mm^2^)
Wall part *	1600	10	0.2	1.875
Capsule-3000	3000	6	0.3	0.400
Capsule-4000	4000	6	0.3	0.300
Capsule-5000	5000	6	0.3	0.240
Capsule-preheating	-	-	-	-

* *The same wall part parameters were used for all the capsules.*

## Data Availability

The data presented in this study are available upon request.

## References

[1] Biamino S., Penna A., Ackelid U., Sabbadini S., Tassa O., Fino P., Pavese M., Gennaro P., Badini C. (2011). Electron beam melting of Ti–48Al–2Cr–2Nb alloy: Microstructure and mechanical properties investigation. Intermetallics.

[2] Wimler D., Lindemann J., Kremmer T., Clemens H., Mayer S. (2021). Microstructure and mechanical properties of novel TiAl alloys tailored via phase and precipitate morphology. Intermetallics.

[3] Appel F., Oehring M., Wagner R. (2000). Novel design concepts for gamma-base titanium aluminide alloys. Intermetallics.

[4] Appel F., Clemens H., Fischer F. (2016). Modeling concepts for intermetallic titanium aluminides. Prog. Mater. Sci..

[5] Todai M., Nakano T., Liu T., Yasuda H.Y., Hagihara K., Cho K., Ueda M., Takeyama M. (2017). Effect of building direction on the microstructure and tensile properties of Ti-48Al-2Cr-2Nb alloy additively manufactured by electron beam melting. Addit. Manuf..

[6] Kim Y.-W., Dimiduk D.M. (1911). Progress in the understanding of gamma titanium aluminides. JOM.

[7] Wu X. (2006). Review of alloy and process development of TiAl alloys. Intermetallics.

[8] Fu Z., Körner C. (2022). Actual state-of-the-art of electron beam powder bed fusion. Eur. J. Mater..

[9] Ladani L., Sadeghilaridjani M. (2021). Review of Powder Bed Fusion Additive Manufacturing for Metals. Metals.

[10] Schwerdtfeger J., Körner C. (2014). Selective electron beam melting of Ti–48Al–2Nb–2Cr: Microstructure and aluminium loss. Intermetallics.

[11] Cho K., Morita N., Matsuoka H., Yasuda H.Y., Todai M., Ueda M., Takeyama M., Nakano T. (2023). Influence of Input Energy Density on Morphology of Unique Layered Microstructure of γ-TiAl Alloys Fabricated by Electron Beam Powder Bed Fusion. Mater. Trans..

[12] Wartbichler R., Clemens H., Mayer S., Ghibaudo C., Rizza G., Galati M., Iuliano L., Biamino S., Ugues D. (2021). On the Formation Mechanism of Banded Microstructures in Electron Beam Melted Ti–48Al–2Cr–2Nb and the Design of Heat Treatments as Remedial Action. Adv. Eng. Mater..

[13] Knörlein J., Franke M.M., Schloffer M., Körner C. (2022). In-situ aluminum control for titanium aluminide via electron beam powder bed fusion to realize a dual microstructure. Addit. Manuf..

[14] Mohammad A., Al-Ahmari A.M., AlFaify A., Mohammed M.K. (2017). Effect of melt parameters on density and surface roughness in electron beam melting of gamma titanium aluminide alloy. Rapid Prototyp. J..

[15] Bieske J., Franke M., Schloffer M., Körner C. (2020). Microstructure and properties of TiAl processed via an electron beam powder bed fusion capsule technology. Intermetallics.

[16] Gao R., Peng H., Guo H., Chen B. (2022). A Combined Powder Metallurgical Approach to Process Gamma-TiAl with Composite Structure. Met. Mater. Trans. A.

[17] Güther V., Allen M., Klose J., Clemens H. (2018). Metallurgical processing of titanium aluminides on industrial scale. Intermetallics.

[18] Aguilar J., Schievenbusch A., Kättlitz O. (2011). Investment casting technology for production of TiAl low pressure turbine blades—Process engineering and parameter analysis. Intermetallics.

[19] Zhu Z.Y., Liu Y.L., Gou G.Q., Gao W., Chen J. (2021). Effect of heat input on interfacial characterization of the butter joint of hot-rolling CP-Ti/Q235 bimetallic sheets by Laser + CMT. Sci. Rep..

[20] Chen Y., Sun S., Zhang T., Zhou X., Li S. (2020). Effects of post-weld heat treatment on the microstructure and mechanical properties of laser-welded NiTi/304SS joint with Ni filler. Mater. Sci. Eng. A.

[21] Wimler D., Lindemann J., Reith M., Kirchner A., Allen M., Vargas W.G., Franke M., Klöden B., Weißgärber T., Güther V. (2021). Designing advanced intermetallic titanium aluminide alloys for additive manufacturing. Intermetallics.

[22] Clemens H., Mayer S. (2013). Design, Processing, Microstructure, Properties, and Applications of Advanced Inter-metallic TiAl Alloys. Adv. Eng. Mater..

[23] Baudana G., Biamino S., Ugues D., Lombardi M., Fino P., Pavese M., Badini C. (2016). Titanium aluminides for aerospace and automotive applications processed by Electron Beam Melting: Contribution of Politecnico di Torino. Met. Powder Rep..

[24] Ghibaudo C., Wartbichler R., Marchese G., Clemens H., Ugues D., Biamino S. (2023). Influence of focus offset on the microstructure of an intermetallic γ-TiAl based alloy produced by electron beam powder bed fusion. J. Manuf. Process..

[25] Liu Y., Li Z., Liu N., Zheng L., Xu W. (2018). Effect of Oxygen Content of Powders on Previous Particle Boundaries in Hot Isostatic Pressed TiAl Alloy. High Performance Structural Materials.

[26] Liu Y., Liang X., Liu B., He W., Li J., Gan Z., He Y. (2014). Investigations on processing powder metallurgical high-Nb TiAl alloy sheets. Intermetallics.

[27] Niu H., Gao T., Sun Q., Zhang H., Zhang D., Liu G. (2018). Prior particle boundaries and microstructural homogenization of a β-solidifying γ-TiAl alloy fabricated from prealloyed powder. Mater. Sci. Eng. A.

